# Regarding effect of different head position during tracheal intubation on postoperative sore throat: a randomized clinical trial

**DOI:** 10.1080/07853890.2025.2541317

**Published:** 2025-07-30

**Authors:** Yu-Fu Guan, Dan-Feng Wang, Zhi-Bin Huang, Fu-Shan Xue

**Affiliations:** a The First Clinical School of Zhengzhou University, Zhengzhou, China; b Department of Anesthesiology, Shengli Clinical Medical College of Fujian Medical University, Fuzhou University Affiliated Provincial Hospital, Fujian Provincial Hospital, Fuzhou, China; c Department of Anesthesiology, Beijing Anzhen Hospital, Capital Medical University, Beijing, China

**Keywords:** Airway management, tracheal intubation, sniffing position, videolaryngoscopy, postoperative sore throat

To the Editor

By conducting a randomized controlled trial in 128 adult patients who underwent elective surgery, Shan and colleagues [1] demonstrated that changing patients’ head position from a sniffing position to a head elevation position during tracheal tube advancement with an angulated videolaryngoscope significantly reduced the incidence of postoperative airway trauma and complications including sore throat and hoarsenes. In their methods, however, Shan et al. [1] did not clearly describe how they placed the patient at a desired sniffing position. According to the part A of figure 1 provided in Shan et al.’ article, we did not agree with them that the participant was rightly placed at the desired sniffing position. The true sniffing position actually contains two components: (a) The neck is flexed 35 degrees on the torso; and (b) the head is extended at the atlanto-occipital joint to produce a angle of 15 degrees between the facial plane and the horizontal plane [2]. Neck flexion aims to approximate the pharyngeal and laryngeal axes, and head extension at the atlanto-occipital joint attempts to align the oral axis with the pharyngeal and laryngeal axes [3]. In clinical practices, the sniffing position is often achieved by placing a non-compressible pad with a height of 5–8 cm under the head and adjusting the operating table headrest to raise the occiput to achieve the neck flexion and head extension at the atlanto-occipital joint. The appropriateness of the sniffing position obtained for each patient can be evaluated by observing the horizontal alignment of the external auditory meatus with the sternal notch [4]. In the sniffing position provided by Shan et al. [1], patient’ neck was actually placed at a significant extension position, rather than the desired flexion position. Both the pad height and head extension at the atlanto-occipital joint were also not enough. In these cases, the horizontal plane through the external auditory meatus is significantly lower than that through the sternal notch and achieving a horizontal alignment of the external auditory meatus with the sternal notch is impossible, indicating an incorrect sniffing position.

Furthermore, according the location and nature of airway trauma, Shan et al. [1] reported categorizations of airway lesions, that is, petechiae, oedema, haematoma, granuloma and others. This only is a toughly qualitative assessment on airway trauma associated with tracheal intubation. In available literature, there have been several scoring systems that can quantitatively assess the severity of airway trauma associated with tracheal intubation [5,6]. We believe that more definitive results of airway trauma severity would have been obtained, if the study by Shan et al. had used one of these scoring systems for comprehensive and quantitative evaluation of airway lesions. Most important, the readers were also not provided if the two groups were comparable with respect to the number of intubation attempt and the occurrence of resistance to tracheal tube advancement, which are the common causes of airway trauma induced by tracheal tube insertion [7]. As a result, it is unclear whether the increased postoperative airway trauma and complications in the sniffing position should really be attributable to difficult tracheal tube advancement.

Finally, Shan et al. [1] did not specify the patient’ status when evaluating postoperative sore throat, though it is less severe at rest than during swallowing [8]. Especially, sufentanil, a powerful opioid drug, was administered for postoperative analgesia by the patient-controlled intravenous analgesia with continuous background infusion and bolus dose. However, Shan et al. [1] did not state if sufentanil consumption in the observed period was comparable between the two groups. Most important, the time relationship between assessment of postoperative sore throat and administration of sufentanil bolus dose was also unclear. It is generally believed that powerful opioid drugs are effective for symptom abatement and treatment of postoperative sore throat. In the absence of these factors, we argue that the main findings of this study must be interpreted with caution, because they would have been obtained by incomplete methodology.

## Data Availability

Data sharing is not applicable to this article as no datasets were generated during current study.
